# Resveratrol Supplementation Did Not Improve Cognition in Patients with Schizophrenia: Results from a Randomized Clinical Trial

**DOI:** 10.3389/fpsyt.2016.00159

**Published:** 2016-09-16

**Authors:** Karine Zortea, Viviane C. Franco, Paula Guimarães, Paulo S. Belmonte-de-Abreu

**Affiliations:** ^1^Schizophrenia Program, Hospital de Clínicas de Porto Alegre, Porto Alegre, Rio Grande do Sul, Brazil; ^2^Postgraduate Program in Medicine, Psychiatry, Universidade Federal do Rio Grande do Sul, Porto Alegre, Rio Grande do Sul, Brazil

**Keywords:** resveratrol, cognition, schizophrenia, nutritional requirements, nutrients, polyphenols

## Abstract

**Background:**

Schizophrenia (SZ) is associated with psychotic experiences and cognitive deficits. Therefore, cognitive function is one of the most critical determinants of quality of life in this pathology. Resveratrol has been related to neuroprotective action, but there are no studies evaluating resveratrol in SZ. The objective of this study was to determine the efficacy of resveratrol supplementation on cognition in individuals with SZ.

**Methods:**

This is a 1-month randomized, double-blind, and controlled trial (NCT 02062190), in which 19 men with diagnosis of SZ, aged 18–65 years, were assigned to a resveratrol supplementation group (200 mg) or placebo group (200 mg), with a 1-month follow-up. Applying a series of cognitive tests assessed neuropsychology performance (Hopkins Verbal Learning Test, Stroop Color and Word Test, and Weschler Adult Intelligence Scale) and Brief Psychiatric Rating Scale assessed psychopathology severity.

**Results:**

There were no significant improvement in neuropsychology performance (episodic memory, working memory, attention and concentration capacity, inhibitory control, interference measures, selective attention, and mental flexibility) and psychopathology severity after 1 month of resveratrol supplementation (*P* > 0.05).

**Conclusion:**

In conclusion, we have shown that 1 month of a resveratrol supplementation (200 mg/day) did not improve episodic memory, working memory, attention and concentration capacity, inhibitory control, interference measures, selective attention, and mental flexibility as compared with placebo in patients with SZ.

## Introduction

Schizophrenia (SZ) is a severe mental disorder characterized by psychotic experiences, and profound disruptions in thinking, affecting language, perception, and the sense of self (1). It is one of the most debilitating psychiatric disorders worldwide (2), and it can impair functioning through the loss of an acquired capability to earn a livelihood or the disruption of studies (1). Individuals with SZ experience a range of cognitive deficits and associated dysfunctions in the neural systems. Deficits of prefrontal and hippocampal systems contribute to disturbances in a number of different cognitive domains, each making different contributions to the nature and severity of cognitive impairments (2). The cognitive–neural system dysfunction may be a key mechanism in the pathway to symptom formation in this pathology and thus plays specific role in the development-specific types of symptoms of SZ. Some cognitive–neural system disturbances may still serve to limit and modify overall life function in individuals with SZ, such as the capacity to function well in their everyday lives. Potential agents for the specific central executive processes impaired in SZ are deficits in context processing and conflict detection, and deficits in recollection than in familiarity processes. These deficits are frequently associated with disruptions in the function or structural integrity of prefrontal cortex, particularly dorsolateral region of prefrontal cortex. There is consistent evidence suggesting impairment in three cognitive domains: working memory, executive control, and episodic memory. Therefore, cognitive function is one of the most critical determinants of quality of life in this pathology (2).

Resveratrol is a polyphenol, produced naturally in dietary sources including grapevines, pines, berries, legumes, and peanuts. It is one of the main agents in the health-promoting effects of red wine, and it can be produced by chemical and biotechnological synthesis as a nutritional supplement (3–5). Resveratrol has been considered the key ingredient responsible for the preventive action of red wine since the stilbene displays a neuroprotective action in various models of toxicity (3, 4). Animal studies have shown that it is able to cross the blood–brain barrier (3).

Studies have been performed to assess the effects of oral resveratrol on cognitive performance in both healthy and cognitively impaired patients (6). These studies provide initial evidence that resveratrol improves memory performance and increases the functional connectivity of the hippocampus in older adults (7). There is mounting evidence that resveratrol exerts neuroprotective effects on neurodegenerative diseases, such as Alzheimer’s, Huntington’s, and Parkinson’s, and that it can protect the brain against the damage induced by toxins and disease (4, 8–10). However, there are no studies evaluating resveratrol in SZ. According to this line of evidence, the objective of this study was to determine the efficacy of resveratrol supplementation on cognition in individuals with SZ.

## Materials and Methods

This is a randomized, double-blind, and placebo-controlled trial of 19 male volunteers, aged 18–65 years, with a diagnosis of SZ established by the Structured Clinical Interview for DSM-IV-Axis I Disorders. Diagnosis of SZ was obtained trough a tree-step method following: (1) clinical interview using information from patient and relatives. (2) Chart review of data and results from routine laboratory exams to check possible clinical differential diagnoses. (3) Final discussion with senior clinical researcher with more than 20 years of work and research in major psychiatric illnesses, for exclusion of differential diagnosis and/or comorbidities. Exclusion criteria were mental retardation and conditions due to alcohol, drug, clinical, or neurological disorders.

The research participants followed a 1-month resveratrol supplementation program, which was covered by the Public Health Service at the SZ Program of Hospital de Clinicas de Porto Alegre (HCPA), Brazil. All participants had been on a stable dose of clozapine (dose range: 100–900 mg) for at least 6 months and provided signed informed consent. Exclusion criteria were the use of other antipsychotic medications or other diagnosis of psychiatric disorder. Participants using antidepressant or anticonvulsant were not excluded. Since this is a preliminary study, no formal sample size calculation was performed.

The subjects were prescribed two dietary supplements a day (200 mg of resveratrol or 200 mg of placebo). Resveratrol (*trans*-resveratrol, 98% purified) and placebo (cornstarch) were obtained from a compounding pharmacy in Porto Alegre, RS, Brazil. The subjects were instructed to take the first supplement after the baseline measurements (day 1) and the last supplement at the end of 1 month (day 30). All assessments were taken on the first and last days (day 1 and day 30) of the 1-month follow-up. They were also instructed to maintain their usual diet and physical activity throughout the study and to abstain from foods containing substantial amounts of resveratrol (e.g., wine, red grapes, peanuts, and berries). They were also advised not to take any other food supplements. The team used two practices to monitor and increase the chances of adherence to the study protocol: (a) weekly telephone calls during the study period and (b) a pill count on the last day (day 30).

A double-blind trial was performed as recorded in the protocol http://clinicaltrials.gov (registration no.: NCT 02062190). The Research Ethics Committee of HCPA approved this research study (registration no.: 110553). CONSORT supported the protocol for this trial.

### Neuropsychology Assessment

Trained psychologists with expertise in psychiatric disorders assessed cognitive performance by applying a series of cognitive tests which took approximately 40 min altogether.

#### Hopkins Verbal Learning Test

This test is meant for individuals 16 years old and older. It evaluates the episodic memory of verbal content, with late and immediate evocation, besides recognition and recall. The test includes a 12-word list with semantic value and a list that adds another 12 words for late recognition. The tasks include three learning trials, one delayed recall trial (20–25 min later), and one late recognition trial (11).

#### Stroop Color and Word Test

This test assesses inhibitory control, interference measures, selective attention, and mental flexibility (12). It is performed in three steps: first, the subjects read a list of color words; then, they name the color of sets of letters; and finally, they name the color of the ink of conflicting color words. This final task requires the subjects to inhibit the urge to read the color word.

#### Weschler Adult Intelligence Scale – Revised

The core objective of the WAIS-R is to provide information that can help identify problem-solving difficulties and specific cognitive deficits (13). The following WAIS-R subtests were used.

#### Digit Span

This subtest involves the oral repetition of numerical sequences in the order they are given (16 items) and the repetition of numerical sequences backwards (14 items), making up 30 points. This subtest investigates immediate repetition (working memory) and recall skills. The task is suspended after two mistakes within the same repetition series.

#### Letter–Number Sequencing

Combinations of numbers and letters are presented to the subject, who in turn should remember the numbers in ascending order and the letters in alphabetic order. Working memory and attention and concentration capacity stand out among the main functions evaluated by this subtest. It has been suggested in the literature that among the measures that compose the Working Memory Index, this subtest is the most sensitive in evaluating neuropsychological deficits resulting from damage to the nervous system.

The Brief Psychiatric Rating Scale (BPRS) assessed psychopathology severity. BPRS evaluates current symptoms and positive and negative dimensions (negative scores refer to the sum of BPRS questions 3, 9, 13, and 16, and positive scores refer to the sum of BPRS questions 8, 11, 12, and 15) (14).

### Statistical Analysis

Statistical analysis was performed using SPSS 17.0 for Windows. The results were represented as the mean ± SD, or median and range, or as percentages (%), as indicated. Data were analyzed by *t*-test (symmetrical distribution) or Mann–Whitney test (asymmetrical distribution) for continuous variables, whereas the Fisher’s exact test was applied for categorical variables. In the pre- and post-intervention comparisons, the paired Student’s *t*-test was applied for normally distributed variables, and the Wilcoxon test was applied for asymmetrical distribution.

## Results

In total, 10 subjects with SZ were included in a resveratrol group and 9 subjects with SZ were placed in a placebo group. All subjects had been on clozapine for at least 6 months. The baseline characteristics of the subject are described in Table 1. The resveratrol and placebo groups were similar in terms of age, education, smoking, and age of onset of the disease. There was a longer length of illness in the resveratrol group, albeit not significant (*P* = 0.07).

**Table 1:** **Baseline clinical parameters of patients in the resveratrol and placebo groups**.

	Resveratrol (*n* = 10)	Placebo (*n* = 9)	*P* value^a^
Age (years)	46.40 ± 11.18	41.00 ± 7.87	0.25
Education (in years)	9.90 ± 3.95	10.22 ± 2.10	0.83
Smoking [*n* (%)]	4 (40)	3 (33)	1.00
Number of cigarette/day	15 (6–20)	30 (20–40)	0.11
Age of onset of the disease (years)	23.90 ± 5.58	26.44 ± 9.67	0.49
Length of illness (years)	22.50 ± 10.00	14.56 ± 7.92	0.07
Clozapine dose (mg/day)	485 ± 213.50	600 ± 180.27	0.23
**Assessment of symptoms**			
BPRS total score	10.5 (2–27)	13 (5–21)	0.72
BPRS positive symptoms	0.5 (0–5)	0 (0–9)	0.97
BPRS negative symptoms	2 (0–14)	3 (0–8)	0.97
**Assessment neuropsychology**			
WAIS-R			
Digits	8.50 (0–13)	7.5 (1–11)	0.76
Letter–number sequences	7.5 (1–12)	6 (4–12)	0.50
Hopkins Verbal Learning Test			
HVLT (total of correct responses)	9.9 ± 2.0	11.2 ± 1.3	0.10
HVLT (total of wrong responses)	2 (0–5)	2 (0–12)	0.78
HVLT (free recall trials)	13.9 ± 4.6	15.2 ± 5.7	0.58
HVLT (delayed recall trial)	43.5 ± 23.1	29.0 ± 11.5	0.11
HVLT (delayed recall trial/time)	24.3 ± 5.7	23.3 ± 11.9	0.86
HVLT (late recognition trial)	7.9 ± 1.7	9.7 ± 2.6	0.10
**Stroop Color and Word Test**			
Stroop (word)	0 (0–7)	0 (0–21)	0.93
Stroop (word–time)	70.1 ± 31.0	58.4 ± 6.6	0.29
Stroop (color)	5 (2–13)	3 (0–8)	0.16
Stroop (color–time)	104.1 ± 16.5	94.6 ± 22.6	0.32
Stroop (word–color)	8 (2–27)	4 (0–13)	0.09
Stroop (word–color–time)	173 (123–285)	117 (0–232)	0.03

*BPRS, Brief Psychiatric Rating Scale; WAIS-R, Weschler Adult Intelligence Scale; HVLT, Hopkins Verbal Learning Test*.

*Data are shown as the mean ± SD, median and range, or relative and absolute frequency*.

*^a^Independent samples *t*-test (symmetrical distribution), Fisher’s exact test (categorical variables), or Mann–Whitney test (asymmetrical distribution). A two-tailed *P*-value <0.05 was considered significant*.

The participants initially formed an apparently homogeneous population regarding all the assessed parameters. Resveratrol group show higher scores in Stroop (word–color–time category) than placebo group, which means that resveratrol group presented worse performance of attention and memory at baseline than placebo group.

Differences between the two groups in the assessed neuropsychology parameters surfaced at the end of 1 month of the trial are showed in Table 2. BPRS, digits, and letter–number sequence were not significantly modified after resveratrol supplementation. In HVLT, delayed recall trial was reduced (*P* = 0.03) in resveratrol group showing worst performance in memory. In the placebo group, we found a significant increase in free recall trials category of HVLT (*P* = 0.05) showing worst performance in memory. In addition, there were no significant side effects.

**Table 2:** **Characteristics of subjects with schizophrenia at baseline and after 1 month of resveratrol supplementation or placebo**.

	Resveratrol			Placebo		
	Day 1 (baseline)	Day 30	*P* value^a^	Day 1 (baseline)	Day 30	*P* value^a^
**Assessment of symptoms**						
BPRS total score	10.5 (2.8–21.3)	16 (3–25.3)	0.48	13 (8–18.5)	14 (4–17.5)	0.92
BPRS positive symptoms	0.5 (0–3.3)	0.5 (0–7)	0.25	0 (0–4)	0.5 (0–2.8)	0.48
BPRS negative symptoms	2 (1.5–8)	3 (0–8.3)	0.93	3 (1–6)	6 (1.5–7.8)	0.20
**Assessment neuropsychology**						
WAIS-R						
Digits	8.5 (0–13)	9 (1–16)	0.17	7.5 (1–11)	8 (2–11)	0.23
Letter–number sequence	7.5 (1–12)	7.5 (3–12)	0.89	6 (4–12)	7 (4–11)	0.29
Hopkins Verbal Learning Test						
HVLT (total of correct responses)	9.9 ± 2.0	9.6 ± 1.6	0.71	11.2 ± 1.3	11.1 ± 0.4	1.00
HVLT (total of wrong responses)	2 (0–5)	2 (0–6)	0.86	2 (0–12)	2.5 (0–12)	0.08
HVLT (free recall trials)	13.9 ± 4.6	16.2 ± 4.9	0.13	15.2 ± 5.7	18.5 ± 3.5	0.05
HVLT (delayed recall trial)	43.5 ± 23.1	24.2 ± 9.6	0.03	29.0 ± 11.5	25.3 ± 4.7	0.45
HVLT (delayed recall trial/time)	24.3 ± 5.7	35.0 ± 14.4	0.23	23.3 ± 11.9	38.4 ± 22.9	0.21
HVLT (late recognition trial)	7.9 ± 1.7	7.4 ± 2.1	0.55	9.7 ± 2.6	10.0 ± 1.8	0.61
**Stroop Color and Word Test**						
Stroop (word)	0 (0–7)	1 (0–9)	0.83	0 (0–21)	0 (0–13)	0.47
Stroop (word–time)	70.1 ± 31.0	68.2 ± 16.6	0.85	58.4 ± 6.6	58.5 ± 9.4	0.68
Stroop (color)	5 (2–13)	3 (1–17)	0.44	3 (0–8)	2.5 (0–16)	0.46
Stroop (color–time)	104.1 ± 16.5	101.0 ± 22.5	0.41	94.6 ± 22.6	100.8 ± 26.2	0.26
Stroop (word–color)	8 (2–27)	8 (0–24)	0.31	4 (0–13)	5 (1–24)	0.39
Stroop (word–color–time)	173 (123–285)	153 (118–313)	0.72	117 (0–232)	152 (93–238)	0.83
Non-adherence (pill returned)	–	5 (0–12)	–	–	9 (2–18)	0.45

*BPRS, Brief Psychiatric Rating Scale; WAIS-R, Weschler Adult Intelligence Scale; HVLT, Hopkins Verbal Learning Test*.

*Data are shown as the mean ± SD or median and range*.

*^a^Paired Student’s t-test was applied for normally distributed variables and Wilcoxon test for asymmetrical distribution. A two-tailed P-value <0.05 was considered significant*.

## Discussion

To our knowledge, this is the first report of a clinical trial with resveratrol supplementation and cognition in patients with SZ. The cognitive tasks assessed episodic memory, working memory, attention and concentration capacity, inhibitory control, interference measures, selective attention, and mental flexibility. In the present data, there were not differences in symptoms (BPRS), working memory and attention (WAIS-R), inhibitory control, interference measures, selective attention, and mental flexibility (Stroop) in resveratrol group as compared to placebo group. HVLT (delayed recall trial and free recall trials category) showed significant differences after resveratrol and placebo supplementation, respectively, which seems to suggest that both resveratrol and placebo group worsened in episodic memory. This result probably occurred in both groups as a consequence of the course of disease (2).

In the last years, resveratrol has received important attention for its anti-tumorigenic, anti-oxidant, and anti-inflammatory benefits. Reports of significant life extension in many *in vitro* and *in vivo* studies supporting a role for resveratrol in treatment of chronic diseases and suggest that resveratrol may have the potential to make a revolutionary impact on human health. Resveratrol has multiple mechanisms of action, such as intrinsic anti-oxidant capacity; interaction with a large number of receptors, kinases, and other enzymes that could plausibly make major contributions to its biological effects; stimulation of the activities of sirtuin-1 and adenosine monophosphate-activated protein kinase, both of which influence the regulation of metabolism in multiple tissues; and others. The complexity of resveratrol’s effects presents a major dare in advance with human studies (15).

The benefits of resveratrol for memory and for prevention of neurodegenerative diseases are well documented in laboratory animals, but this aspect is only beginning to be explored through a number of ongoing human clinical trials (15). Thus, there are conflicting results as to the effect of resveratrol in humans, with some studies reporting improvements while others find no effects (7, 16). Kennedy et al. (6) studied the vasodilatory action of resveratrol in a placebo-controlled study involving 22 healthy adults and showed that administration of resveratrol (250 and 500 mg) resulted in dose-dependent increases in cerebral blood flow during cognitive task performance that activates the frontal cortex. Nevertheless, cognitive functions were not affected. In a similar study, Wightman et al. performed a randomized, double-blind, and placebo-controlled, with 60 healthy adults receiving placebo or resveratrol for 28 days. At the end of the study, the performance of cognitively demanding tasks did not result in any clear improvements in cognitive function. Previous study shows that the lack of cognitive effects may be due to low resveratrol bioavailability and it reduced efficacy *in vivo* (17). The bioavailability of polyphenols is a complex process influenced by several factors such as food composition, dietary patterns, and nutritional and pathophysiological status of individuals (8).

Interesting, Witte et al. (7) studied 23 healthy overweight older individuals that completed 26 weeks of resveratrol intake (200 mg/day) and provided initial evidence that supplementary resveratrol improve memory performance. It supports the hypothesis that chronic resveratrol ingestion may exert positive effects on brain function. Possibly, beneficial effects on brain may translate into behavioral improvements after a sufficiently longer duration of resveratrol intake.

Since the cognitive–neural system dysfunction may be a key mechanism in the pathway to symptom formation in SZ and resveratrol supplementation has demonstrated neuroprotective action, we hypothesized that resveratrol could improve the symptoms of psychiatric disorders. Unfortunately, current symptoms and positive and negative dimensions were not significantly modified after resveratrol supplementation in this study.

There are many reasons for resveratrol’s lack of efficacy in human studies. First, there is a considerable heterogeneity in study quality, design, and polyphenol formula/dosage (16). Second, the optimal resveratrol dose, timing, and duration are unknown. Third, resveratrol has relatively low bioavailability due to its substantial and rapid hepatic metabolism. Another possible explanation is the fact that it is the total polyphenols in red wine, not resveratrol alone, that generate the beneficial effects of red wine (18). And finally, it is difficult to establish good adherence to treatment in patients with psychiatric disorders (19).

It is important to point out that this study must be considered in light of some limitations: the study used a small sample size, specially for outcome measures such as cognitive performance, which ideally require a larger sample than the physiological measures; a crossover method was not included; and the study used a 1-month supplementation period, which could be deemed short. This study provides initial results about resveratrol supplementation on cognitive performance, but future long-term clinical trials (greater than 6 months) with larger sample sizes are needed to consider possible neuroprotective mechanisms of resveratrol.

## Conclusion

In conclusion, we have shown that 1 month of a resveratrol supplementation (200 mg/day) did not improve episodic memory, working memory, attention and concentration capacity, inhibitory control, interference measures, selective attention, and mental flexibility as compared with placebo in patients with SZ.

## Author Contributions

KZ wrote the protocol, designed the study, and participated in data acquisition and interpretation, in drafting the article, and in securing the final approval of this version. VF and PG participated in the study design, data acquisition, and interpretation. PSBA designed the study, wrote the protocol, and was responsible for interpreting the data, drafting the article, and obtaining the final approval of this version. All authors contributed to the final version of the manuscript.

## Conflict of Interest Statement

The authors declare that the research was conducted in the absence of any commercial or financial relationships that could be construed as a potential conflict of interest.
